# Data-Driven Object Vehicle Estimation by Radar Accuracy Modeling with Weighted Interpolation †

**DOI:** 10.3390/s21072317

**Published:** 2021-03-26

**Authors:** Woo Young Choi, Jin Ho Yang, Chung Choo Chung

**Affiliations:** 1 Departerment of Electrical Engineering, Hanyang University, Seoul 04763, Korea; wooyoungchoi@hanyang.ac.kr (W.Y.C.); jjz0426@hanyang.ac.kr (J.H.Y.); 2 Division of Electrical and Biomedical Engineering, Hanyang University, Seoul 04763, Korea

**Keywords:** object vehicle estimation, radar accuracy, data-driven, radar latency, weighted interpolation, autonomous vehicle

## Abstract

For accurate object vehicle estimation using radar, there are two fundamental problems: measurement uncertainties in calculating an object’s position with a virtual polygon box and latency due to commercial radar tracking algorithms. We present a data-driven object vehicle estimation scheme to solve measurement uncertainty and latency problems in radar systems. A radar accuracy model and latency coordination are proposed to reduce the tracking error. We first design data-driven radar accuracy models to improve the accuracy of estimation determined by the object vehicle’s position. The proposed model solves the measurement uncertainty problem within a feasible set for error covariance. The latency coordination is developed by analyzing the position error according to the relative velocity. The position error by latency is stored in a feasible set for relative velocity, and the solution is calculated from the given relative velocity. Removing the measurement uncertainty and latency of the radar system allows for a weighted interpolation to be applied to estimate the position of the object vehicle. Our method is tested by a scenario-based estimation experiment to validate the usefulness of the proposed data-driven object vehicle estimation scheme. We confirm that the proposed estimation method produces improved performance over the conventional radar estimation and previous methods.

## 1. Introduction

Autonomous driving technologies such as collision risk decision, path planning with collision avoidance, lane change systems, and advanced driver assistance systems (ADASs) are attracting attention [1,2,3,4]. These research areas are becoming critical not only for research but also to bring autonomous vehicles to public roads. To improve active safety systems for autonomous driving, it is necessary to accurately estimate the relative position of surrounding vehicles [5,6]. Object vehicle estimation research incorporates various types of sensors, such as radio detecting and ranging (radar), light detection and ranging (LiDAR), and cameras. Among the various sensors, radar is a reliable vehicle sensor that measures the motion of surrounding vehicles. Its advantages lie in its commercial availability and robustness against environmental variation. Radar sensors have been applied in ADASs functions such as blind-spot detection (BSD) and adaptive cruise control (ACC).

However, radar has intrinsic measurement uncertainties in calculating an object vehicle’s position and velocity as it uses a virtual polygon box with only partial information [7,8,9]. To address this limitation, various filters have been applied to improve radar accuracy. In radar applications, the Kalman filter (KF) and the interacting multiple model (IMM) were compared in [10]. A particle filter [11] and an unscented Kalman filter (UKF) [12] for nonlinear systems have been proposed for target tracking using a radar sensor. For reasonable object tracking of a radar system, it has been found that the multiple model approach provides better filtering performance than a single model [13]. Radar tracking performance is improved through IMM [14] and convex interpolation [15] by using different radar accuracies depending on the object vehicle position [16]. In [9], the authors proposed an IMM algorithm using extended Kalman filters (EKF) for multi-target state estimation. In [17], the performances of the IMM and Viterbi algorithm were investigated and compared through radar tracking and detection. A self-adapting variable structure multiple model (VS-IMM) estimation approach combined with an assignment algorithm was presented in [18] for tracking ground targets with constrained motion. Motion uncertainties due to variable dynamic driving situations were handled using the VS-IMM. In [19], the authors presented a data-driven object tracking approach by training a deep neural network to learn situation-dependent sensor measurement models.

Another approach to accurate object tracking using radar adds sensors such as a camera and LiDAR. Research fusing radar and camera sensors is described in [20]. In [21], the authors used visual recognition information to improve tracking model selection, data association, and movement classification. An algorithm to estimate the location, size, pose, and motion information of a threat vehicle was implemented by fusing the information from a stereo-camera and from millimeter-wave radar sensors in [22]. In [23], the authors proposed a fusion architecture using radar, LiDAR, and camera for accurate detection and classification of moving objects. In [24], heuristic fusion with adaptive gating and track to track fusion were applied to a forwarding vehicle tracking system using camera and radar sensors, and the two algorithms were compared. In [25], the authors presented an EKF that reflects the distance characteristics of LiDAR and radar sensors. In [26], the fusion of radar and camera sensor data with a neural network was studied to improve object detection accuracy. In [27], the object was identified and detected using vision and radar sensor data, and YOLOv3 architecture. However, the sensor fusion approach requires a larger number of sensors. Although the estimation performance can be improved through multi-sensor applications, it increases the vehicle’s cost. In addition, latency occurs due to the increase in computational cost for sensor fusion [28].

As stated above, by applying a filter without an additional sensor, accurate tracking is possible without increasing the cost. However, radar latency (processing delay) increases with the use of a filter [16,29,30]. This latency increases further depending on the tracking algorithm used (e.g., point cloud clustering, segmentation, single sensor tracking, multilateration, classification, and filtering) in vehicle applications [7,14,31,32]. In this regard, the radar sensor was evaluated for the effect of processing latency on the efficiency of detecting, acquiring, and tracking a target [29]. In [33], the authors noted that it is important for delays in the measurement (i.e., the time elapsed since a physical event occurs until it is output to the application) and accurate data on the position of other vehicles in future driver assistance systems. In [34], the authors proposed a classification method based on deep neural networks using automotive radar sensors in consideration of latency. Eventually, this processing latency causes a tracking error depending on the relative speed in autonomous driving applications. Therefore, a person who designing an upper-level application should consider processing latency when developing object vehicle estimation for driving safety.

The objective of this paper is to propose an object vehicle estimation scheme to improve radar accuracy. The scheme develops a data-driven object vehicle estimation scheme that can consider radar accuracy within a feasible set to solve the measurement uncertainty and latency problems. To resolve these problems, we first develop radar accuracy models by comparing the radar and ground truth data divided in each zone. Each zone’s models are selected depending on where the object vehicle is located. We then solve the radar latency problem according to the relative velocity. The position error for the relative velocity data sets is stored in each vertex, and we find the solution in the feasible set for these data sets. By using the developed radar accuracy models with latency coordination, weighted interpolation is applied to estimate the object vehicle. This approach will allow the radar accuracy models to remove the measurement uncertainty and latency within the feasible set. We verify the utility of the proposed method through scenario-based experiments. The contribution of this paper is the developmetn of an accurate object vehicle estimation scheme that solves the radar measurement uncertainty and latency problems.

The remainder of this paper is organized as follows. Section 2 describes two problems to improve object vehicle estimation accuracy. The data-driven radar accuracy modeling with an occupancy zone is described in Section 3. In Section 4, weighted interpolation is applied to object estimation by considering error characteristics and latency. Section 5 describes the analysis and results by applying the proposed method to vehicle applications and mentions future work. Section 6 presents concluding remarks.

## 2. Problem Statement

**Figure 1:**
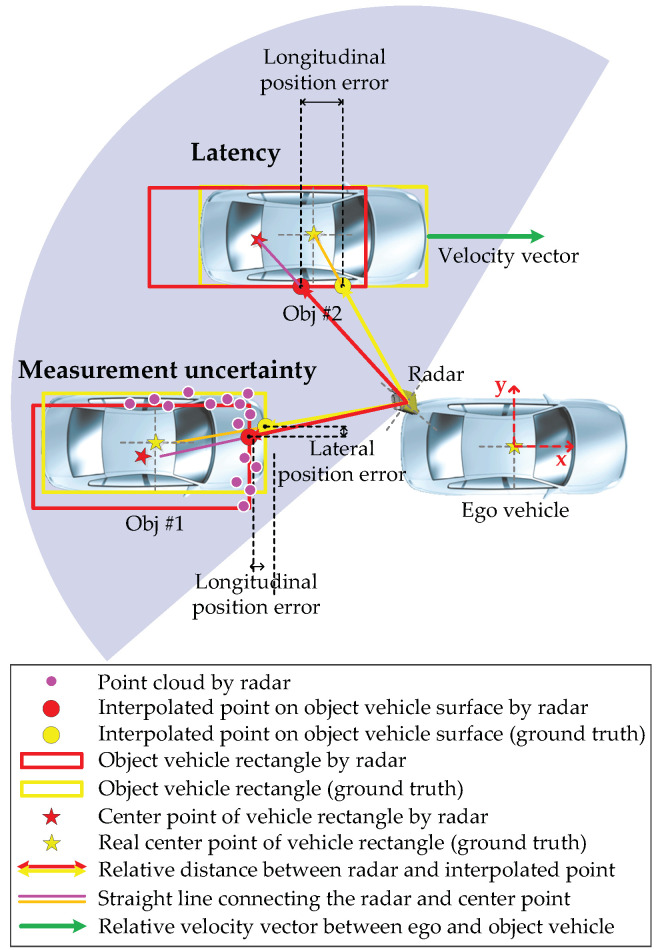
Example of object vehicle estimation by radar: the measurement uncertainty occurs due to insufficient point cloud and classification errors. This error occurs because the radar estimation algorithm (e.g., point cloud clustering, segmentation, single sensor tracking, multilateration, classification, and filtering) can only estimate an object vehicle’s size with a virtual polygon box with partial information [7,8,14,35]. Furthermore, the latency that causes position errors occurs due to the tracking algorithm of a commercial radar. The error caused by the latency becomes larger depending on the relative velocity.

The problem we are interested in is object vehicle estimation by considering a radar’s measurement uncertainty and latency, as shown in Figure 1. There are two fundamental problems in accurate object vehicle estimation: measurement uncertainties in calculating an object’s position with a virtual polygon box and latency due to the tracking algorithm of a commercial radar. To resolve these problems, we develop a data-driven object vehicle estimation scheme using a radar accuracy modeling method with weighted interpolation. The radar accuracy modeling is designed using an error model between the radar and the ground truth data, and taking into account the relative speed. We are also interested in demonstrating the utility of our method through experiments.

These problems are almost undetectable and unknown to those who develop high-level applications such as ADASs. Therefore, we propose a scheme modeling these undetectable and unknown error characteristics as noise characteristics based on each divided zone and design a data-driven object estimation scheme. In addition, we propose a method to reduce errors that occur in radar algorithms by developing data-driven latency coordination. We use the relative position and velocity, which are the only available data to the person designing an upper-level application.

## 3. Data-Driven Radar Accuracy Modeling

To improve radar accuracy, we first developed a model. Previous research analyzing radar accuracy [14,15] found that the error characteristics differ according to the mounting angle and detection area of the radar observing the object [16]. Since the radar error differs depending on the angle and the detection area, it is difficult to obtain an error characteristic solution for a radar’s detection area. Therefore, we model these unknown error characteristics so that an error has the same value in each of the divided representative detection zones because the part of the object vehicle detected by the radar is similar to other object vehicles in the same detection area. In other words, each radar unit has a representative model for each zone. The measurement uncertainty of radar can be reduced by using an occupancy zone with the error characteristics. However, there is an error according to the object vehicle’s velocity due to the radar’s latency (the results of the analysis of the experimental data are shown in Section 4). This is caused by the object tracking algorithm of commercial radars [14,29]. This is a problem for anyone designing high-level applications for radar.

**Figure 2:**
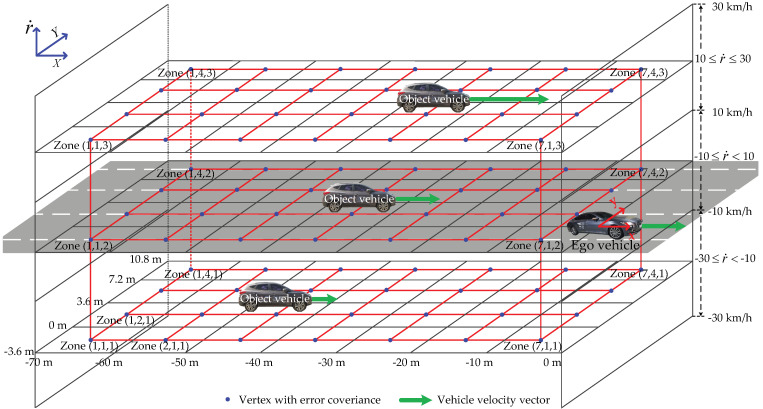
Example of a divided occupancy zone configuration: the occupancy zone is created by taking into account the detectable area of the radar, which is divided by the x-axis and y-axis based on the vehicle coordinate frame. The z-axis is divided by data sets for relative velocity. The center point of each divided black quadrangle zone becomes each vertex of the red quadrangle (feasible set for error covariance). Then, the error characteristics analyzed in each zone are stored in each vertex.

Therefore, we constructed an example of occupancy zones, as shown in Figure 2, taking into account the detectable area of the radar, where {X,Y} is the global coordinate frame, {x,y} is the ego vehicle coordinate frame, and $\dot{r}$ is the relative vehicle speed. The example of a divided occupancy zone configuration is divided by the *x*-axis (considering a multiple of the overall vehicle length), the *y*-axis (considering lane spacing), and the *z*-axis (considering experimental data analysis) based on the vehicle coordinate frame. Here, the *z*-axis is divided by data sets for each relative speed. The center point of each divided black quadrangle zone becomes each vertex of the red quadrangle (feasible set for error covariance). Then, the error characteristics analyzed in each zone are stored in each vertex. The radar accuracy in each divided occupancy zone detected by radar sensors is analyzed by comparing radar sensor data with ground truth (GT), as shown in Figure 1. An interpolated point on the object vehicle surface is calculated by a straight line connecting the ego vehicle’s radar and the center point of the virtual polygon box of the object vehicle. Here, the real center point (ground truth) is calculated from the differential global positioning system (DGPS) mount point. Then, we can obtain the longitudinal and lateral position errors by comparing the interpolated point and the center point.

The model for object vehicle estimation can be expressed as a discrete-time state-space model assuming that the vehicle is moving with constant relative velocity in the longitudinal and lateral directions, respectively [36]. With the state $x_{k}={\left[\begin{array}{cccc} r_{x} & r_{y} & {\dot{r}}_{x} & {\dot{r}}_{y} \end{array}\right]}^{T}$, the state-space model is defined as

(1) $$\begin{array}{cc} x_{k+1} & =\Phi x_{k}{+}_{\left(m,n,s\right)}\;\;w_{k},\\ y_{k} & =Cx_{k}{+}_{\left(m,n,s\right)}\;\;v_{k} \end{array}$$

where

(2) $$\begin{array}{cc} \Phi =\left[\begin{array}{cccc} 1 & T_{c} & 0 & 0\\ 0 & 1 & 0 & 0\\ 0 & 0 & 1 & T_{c}\\ 0 & 0 & 0 & 1 \end{array}\right] & ,\\ \;{\;}_{\left(m,n,s\right)}\;w_{k}∼N(0{,}_{\left(m,n,s\right)} & \;Q_{k}),\\ \;{\;}_{\left(m,n,s\right)}\;v_{k}∼N(0{,}_{\left(m,n,s\right)} & \;R_{k}) \end{array}$$

with

(3) $$\begin{array}{c} m\in [1,\;2,\;\cdots ,\;M],\;n\in [1,\;2,\;\cdots ,\;N],\;s\in [1,\;2,\;\cdots ,\;S] \end{array}$$

where $y_{k}$ is the output variables at the measurement instant *k*, ${}_{\left(m,n,s\right)}\;w_{k}$ is the system noise, ${}_{\left(m,n,s\right)}\;v_{k}$ is the radar measurement accuracy, *C* is the identity matrix, $r_{x}$ is the longitudinal relative distance, $r_{y}$ is the lateral relative distance, ${\dot{r}}_{x}$ is the longitudinal relative velocity, ${\dot{r}}_{y}$ is the lateral relative velocity, *m* is the longitudinal relative positional zone index, *n* is the lateral relative positional zone index, *s* is the zone index for relative velocity, *M* is the zone number of the *X*-axis, *N* is the zone number of the *Y*-axis, and *S* is the zone number of the *Z*-axis.

**Assumption** **1.** *Radar measurement accuracy ${}_{\left(m,n,s\right)}\;v_{k}$ has a zero-mean white Gaussian distribution property in each zone [37]. The radar measurement accuracy covariance ${}_{\left(m,n,s\right)}R_{k}$ is a value determined by the characteristics of the sensor. The radar measurement accuracy covariance ${}_{\left(m,n,s\right)}R_{k}$ in each zone is set based on the error characteristics. The radar sensor is calibrated at each zone, such that the mean value of the position error becomes zero. Therefore, the zero-mean radar error becomes*

(4) $$\begin{array}{c} e={\left[\begin{array}{c} r_{x},\;r_{y},\;{\dot{r}}_{x},\;{\dot{r}}_{y} \end{array}\right]}_{RADAR}^{T}-{\left[\begin{array}{c} r_{x},\;r_{y},\;{\dot{r}}_{x},\;{\dot{r}}_{y} \end{array}\right]}_{GT}^{T} \end{array}$$

*and its covariance is*

(5) $$\begin{array}{c} E\left[\begin{array}{c} ee^{T} \end{array}\right]∼N\left(0{,}_{\left(m,n,s\right)}\;R_{k}\right) \end{array}$$

*where subscript GT represents the ground truth data and subscript RADAR represents the calibrated radar data. Since it is not easy to obtain radar accuracy covariance values according to driving situations, we experimentally applied covariance values based on the method presented in [38]. In this regard, the experimental analysis results with the calibrated radar accuracy are shown in Section 4.*

**Remark** **1.** *By adjusting the system noise covariance ${}_{\left(m,n,s\right)}Q_{k}$ through the KF in which the previously set radar measurement accuracy covariance ${}_{\left(m,n,s\right)}R_{k}$ is used, estimation errors approaching the minimum value in each zone are obtained [39]. Then, we set the system noise covariance ${}_{\left(m,n,s\right)}Q_{k}$ for each zone.*

## 4. Object Tracking with Weighted Interpolation

### 4.1. Estimation with Error Characteristic

The weighted interpolation in the occupancy zone is applied to state estimation by considering the error characteristics. The weighted interpolation method is used to solve the ambiguity problem of moving from a zone to another zone caused by dividing the occupancy zone. To apply weighted interpolation, we create a feasible set $f_{c}$ denoted by a red quadrangle relative to the center point of each black quadrangle zone in Figure 2. The center point of each zone is the vertex of the feasible set $f_{c}$ for error covariance, and $f_{c}$ takes into account the lane width. The data-driven covariance $R^{*}$ calculated in the previous section is stored at each zone’s vertex. This process is carried out offline using data analyzed in advance.

In online computation, the object vehicle positions x and y, and relative speed $\dot{r}$ are given by the radar sensor. Then, the data-driven covariance stored at the vertex is applied to state estimation. The three-dimensional parameter vector $P_{c}={\left[\begin{array}{ccc} x & y & \dot{r} \end{array}\right]}^{T}\in R^{3}$ can be represented in the polytopic form [15,40,41]:

(6) $$\begin{array}{cc} P_{c} & =V_{c}\xi_{c} \end{array}$$

where

(7) $$\begin{array}{c} \xi_{c}={\left[\begin{array}{ccc} \xi_{c,1} & \cdots & \xi_{c,8} \end{array}\right]}^{T}\in R^{8} \end{array}$$

denotes a weighted interpolation parameter vector satisfying $\sum_{q=1}^{8}\xi_{c,q}=1$, $\xi_{c,q}\geq 0$, and

(8) $$\begin{array}{c} V_{c}=\left[\begin{array}{ccc} P_{c,1} & \cdots & P_{c,8} \end{array}\right]\in R^{3\times 8} \end{array}$$

denotes each zone’s vertices. When selecting the each zone’s vertices $V_{c}$, we chose the eight vertices closest to the given x, y, and $\dot{r}$ measured by the radar in the feasible set $f_{c}$, as shown in Figure 2. Then, we can get

(9) $$\begin{array}{c} \xi_{c,q}=\frac{L_{c,sum}/L_{c,q}}{\sum_{i=1}^{8}\left(L_{c,sum}/L_{c,i}\right)},\;q=1,\cdots ,8 \end{array}$$

where $L_{c,sum}=\sum_{i=1}^{8}L_{c,i}$ in which $L_{c}$ is the Euclidean distance between each vertex and the given point (x, y, $\dot{r}$) measured by the radar. Using the interpolation parameters with the parameter vector at eight vertices, we can find an approximate data-driven covariance $R^{o}$ from the precomputed data-driven covariance $R^{*}\left(V_{c}\right)$ calculated from Assumption 1 at each vertex. The approximate data-driven covariance $R^{o}$ is expressed as follows:

(10) $$\begin{array}{c} R^{o}=\left[\begin{array}{c} R^{*}\left(P_{c,1}\right)\;\cdots \;R^{*}\left(P_{c,8}\right) \end{array}\right]\xi_{c}. \end{array}$$

From the KF using $R^{o}$, we can obtain the estimated object vehicle position $\hat{x}$ and $\hat{y}$ [39,42]. This approach satisfies the computational complexity because it does not consider all the zones’ vertices. Here, we describe covariance related to the position for the object vehicle estimation; the covariance related to the velocity can be referred to [14] in a similar way.

### 4.2. Latency Coordination

To solve the aforementioned latency problem, weighted interpolation is applied to the state estimation similar to the previous subsection. As stated above, a radar’s latency varies depending on the relative velocity. The velocity region is divided, and the average position error by latency that occurred in each velocity set is stored in each vertex. Detailed data analysis is provided in Section 4.

The object vehicle longitudinal relative velocity ${\dot{r}}_{x}$ and lateral relative velocity ${\dot{r}}_{y}$ are given by the radar sensor. Then, the position error average $E_{l}^{*}$ by latency in each velocity region stored at the vertex is applied to the state estimation. Two-dimensional parameter vector $P_{l}={\left[\begin{array}{cc} {\dot{r}}_{x} & {\dot{r}}_{y} \end{array}\right]}^{T}\in R^{2}$ can be represented in the polytopic form:

(11) $$\begin{array}{cc} P_{l} & =V_{l}\zeta_{l} \end{array}$$

where

(12) $$\begin{array}{c} \zeta_{l}={\left[\begin{array}{cc} \zeta_{l,1} & \zeta_{l,2} \end{array}\right]}^{T}\in R^{2} \end{array}$$

denotes the weighted interpolation parameter vector satisfying $\sum_{p=1}^{2}\zeta_{l,p}=1$, $\zeta_{l,p}\geq 0$, and

(13) $$\begin{array}{c} V_{l}=\left[\begin{array}{cc} P_{l,1} & P_{l,2} \end{array}\right]\in R^{2\times 2} \end{array}$$

denotes the vertices. When selecting two vertices from the given relative velocities ${\dot{r}}_{x}$ and ${\dot{r}}_{y}$ measured by the radar, we chose two vertices that matched the relative velocity data set from the viable set $f_{l}$. Then, we can get

(14) $$\begin{array}{c} \zeta_{l,p}=\frac{L_{l,sum}/L_{l,p}}{\sum_{j=1}^{2}\left(L_{l,sum}/L_{l,j}\right)},\;p=1,2 \end{array}$$

where $L_{l,sum}=\sum_{j=1}^{2}L_{l,j}$ in which $L_{l}$ is the Euclidean distance between each vertex and the given relative velocities point (${\dot{r}}_{x}$, ${\dot{r}}_{y}$) measured by the radar. Using the interpolation parameters given the parameter vector for the relative velocity at two vertices, we can find an approximate position error from the interpolation between the precomputed average position error $E_{l}^{*}\left(V_{l}\right)\in R^{2\times 2}$ at each vertex. The approximate position error $E_{l}^{o}\in R^{2}$ is expressed as follows

(15) $$\begin{array}{c} E_{l}^{o}=\left[\begin{array}{cc} E_{l}^{*}\left(P_{l,1}\right) & E_{l}^{*}\left(P_{l,2}\right) \end{array}\right]\zeta_{l}. \end{array}$$

Using the precomputed position error average $E_{l}^{*}\left(V_{l}\right)$ at each vertex, we can calculate the approximate position error $E_{l}^{o}={\left[\begin{array}{cc} x^{o} & y^{o} \end{array}\right]}^{T}$ for a given relative velocity ${\dot{r}}_{x}$ and ${\dot{r}}_{y}$. The approximate position error $E_{l}^{o}$, and $\hat{x}$ and $\hat{y}$ calculated by KF in the previous subsection are directly involved in the determination of estimated approximate position ${\hat{x}}^{o}$ and ${\hat{y}}^{o}$:

(16) $$\begin{array}{c} {\hat{x}}^{o}=\hat{x}-x^{o},\;\;{\hat{y}}^{o}=\hat{y}-y^{o}. \end{array}$$

Then, estimated approximate position ${\hat{x}}^{o}$ and ${\hat{y}}^{o}$ are applied to object vehicle tracking.

## 5. Application

We experimentally validated how useful the proposed data-driven weighted interpolation algorithm is when applied to object vehicle estimation of an autonomous vehicle.

### 5.1. Experimental Setup

**Figure 3:**
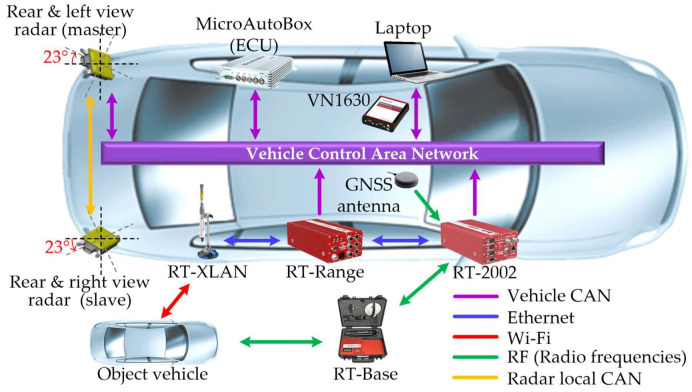
Hardware configuration of the experimental setup.

**Figure 4:**
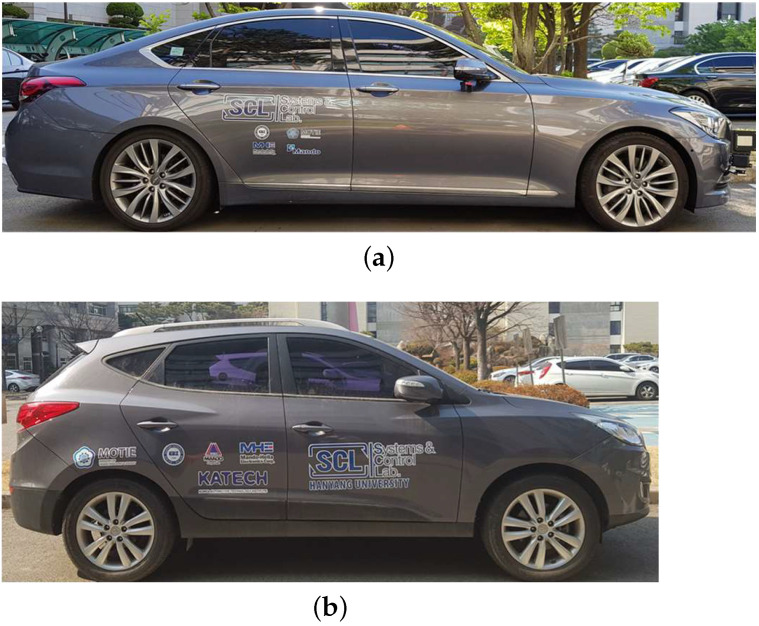
Vehicles used for experiment: (**a**) ego vehicle: Genesis DH from Hyundai and (**b**) object vehicle: Tucson IX from Hyundai.

For the experimental setup shown in Figure 3, the ego and object vehicles used were Genesis DH and Tucson IX from Hyundai, as shown in Figure 4, respectively. The rear left, and rear right view radars connected by a master and slave system with radar local control area network (CAN) were located on both sides of the rear of the ego vehicle and were rotated 23 degrees outward.

**Figure 5:**
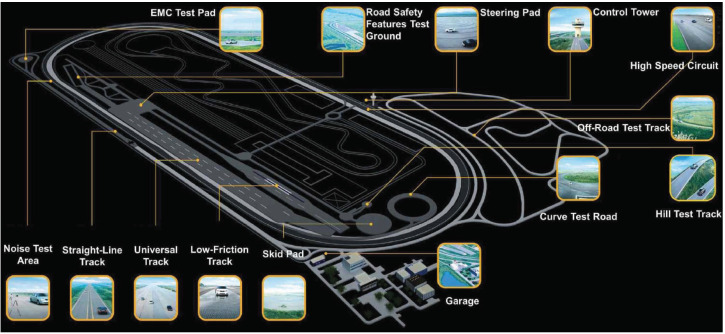
Test road: Korea Automobile Testing & Research Institute (KATRI).

The radar used 24 GHz BSD from Mando-Hella Electronics Corp., in Incheon, South Korea, the update sampling rate was 50 ms, and the distance detect range was up to 70 m. The ground truth data were collected at an update period of 10 ms using DGPS from OxTS (RT-2002, RT-Range, global navigation satellite system (GNSS) antenna, RT-XLAN, and RT-Base) with its real-time kinematic (RTK) positioning service (1σ = 0.01 m). We collected the object vehicle’s ground truth data through the RT-Range and RT-XLAN Wi-Fi. Radar and DGPS data were collected through MicroAutoBox from dSPACE, analyzed with Vector’s CANoe with VN1630, and evaluated using MATLAB/Simulink. These data were given by the ego and object vehicle driven manually on a high-speed circuit in the Korea Automobile Testing & Research Institute (KATRI) in South Korea, as shown in Figure 5.

### 5.2. Radar Accuracy Analysis

**Figure 6:**
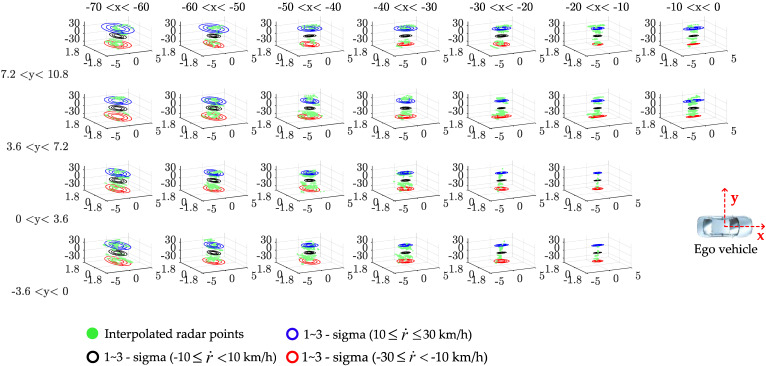
Data-driven radar accuracy analysis in the divided occupancy zone: each zone shows the probability density function contour (1 σ, 2 σ, and 3 σ) of the normal distribution for radar’s position error based on differential global positioning system (DGPS). Contour lines are shown in blue, black, and red for each speed data set.

The radar accuracy was analyzed by the occupancy zone, as shown in Figure 6. The radar accuracy was analyzed by comparing the DGPS and radar data in each divided occupancy zone. Each zone shows the probability density function contour of the normal distribution based on DGPS, and blue (10 $\leq \dot{r}\leq$ 30 km/h), black ($-10\;\leq \dot{r}<$ 10 km/h), and red ($-30\;\leq \dot{r}<\;-$10 km/h) colors were plotted for each speed data set. It was found that the radar accuracy was different depending on the relative distance and speed. The error was increased as the relative distance and relative velocity between the ego vehicle and the object vehicle increased. Depending on the relative distance, measurement uncertainties by radar occurred [14,16]. We collected data through various real driving situations. The radar accuracy analysis was based on a total of 193,324 samples In this regard, the longitudinal relative velocity between the two vehicles was about −30 to 30 km/h.

**Remark** **2.** *If the amount of sampled calibrated sensor data increases, the distribution of the measurement noise becomes the Gaussian distribution, as shown in Figure 6. Therefore, the system has better performance with more calibrated sensor data. Here is a reference if the measurement noise is not Gaussian [43].*

**Figure 7:**
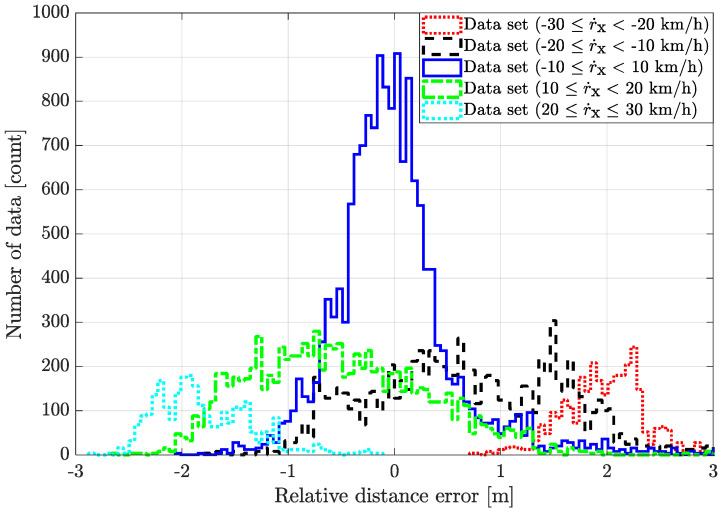
Histogram of the radar relative distance error depending on the vehicle speed.

The longitudinal position error, which increases with relative velocity, was due to the latency of the radar, as shown in Figure 7. The average position error $E_{l}^{*}$ of each velocity data set was analyzed as follows:

(i) The average position error of the data set ($-30\leq {\dot{r}}_{x}<-20$ km/h) is 1.968 m.
(ii) The average position error of the data set ($-20\leq {\dot{r}}_{x}<-10$ km/h) is 0.709 m.
(iii) The average position error of the data set ($-10\leq {\dot{r}}_{x}<$ 10 km/h) is 0.018 m.
(iv) The average position error of the data set (10 $\leq {\dot{r}}_{x}<$ 20 km/h) is −0.511 m.
(v) The average position error of the data set (20 $\leq {\dot{r}}_{x}\leq$ 30 km/h) is −1.793 m.

The calculated position error average by latency was stored at each vertex of the velocity regions. Based on the analysis results, the position error and covariance values of each zone’s error were obtained for the filter design.

The point to note here is that Figure 6 and Figure 7 showed different results from what is generally understood, and therefore, careful attention is required. The radar’s point cloud data is accurate in the longitudinal direction and inaccurate in the lateral direction. This is certain in radar’s row data. However, providing a cloud data point to users makes it difficult for upper-level users to use radar data. Therefore, commercial radar represents an object as one tracking point data through an estimation algorithm (e.g., point cloud clustering, segmentation, single sensor tracking, multilateration, classification, and filtering) [7,8,14,35]. Therefore, there is latency (processing delay). The greater the difference in speed between the ego vehicle and the object vehicle, the greater the latency, and in a vehicle application with a velocity in the longitudinal direction, it causes a longitudinal error. As a result of this, unlike the general idea that the relative longitudinal distance of the radar is more accurate than the relative lateral distance, the experimental data with DGPS shows that the longitudinal direction is more inaccurate than the lateral direction. This means that, as the relative speed increases, the longitudinal error increases. Therefore, anyone designing an upper-level application needs to increase the radar accuracy. This is why we used object vehicle estimation with radar accuracy modeling.

**Remark** **3.** *The latency of the relative lateral velocity is insignificant so it is not considered [44]. It can be calculated similarly to the method calculating the position error by latency.*

### 5.3. Scenario-Based Experimental Result

An object vehicle tracking scenario is constructed using data-driven object vehicle estimation with a radar sensor. For a comparative study of object vehicle tracking, we collected radar data while the object vehicle was driving in the detectable area of the rear left radar of the ego vehicle. As stated above, we determined the error characteristics in the occupancy zone by analyzing radar accuracy. Then, the approximate object estimation data were obtained by the data-driven weighted interpolation process using error characteristics data. When using weighted interpolation, the interpolation parameter vector was designed to satisfy $0<\epsilon \leq \xi_{c,q}$ and $0<\varepsilon \leq \zeta_{l,p}$ for numerical stability, where ϵ and ε are small values. The proposed process improves the estimation performance of the commercial radar and the previously studied interpolation method [15].

**Figure 8:**
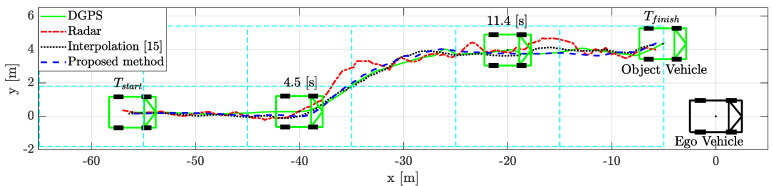
Scenario-based relative movement of object estimation from the top view: when estimated with a commercial radar, the longitudinal and lateral position errors (between −37 and −33 m) and lateral position error (between −17 and −15 m) occurred due to latency and measurement uncertainty.

**Figure 9:**
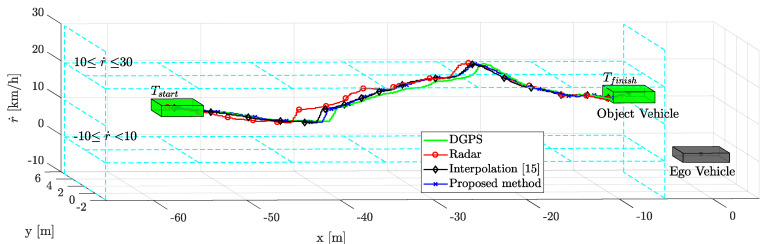
Scenario-based relative movement of object estimation with relative speed in a 3D view.

**Figure 10:**
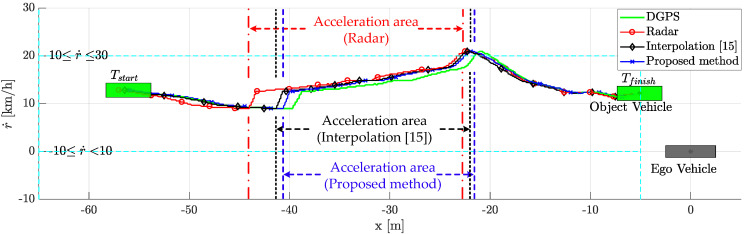
Scenario-based relative movement for relative longitudinal distance and relative speed replotted from Figure 9: there is an acceleration area because the object vehicle changes lanes with increasing speed to overtake the ego vehicle. After that, the relative speed decreases.

**Figure 11:**
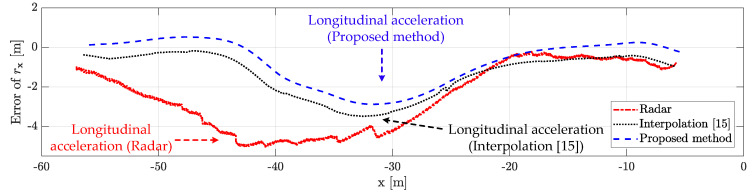
Longitudinal distance error for scenario-based object estimation: the proposed method outperforms the conventional radar estimation method and the previous interpolation method. However, there is a longitudinal error in all methods due to latency for longitudinal acceleration in the acceleration area.

**Figure 12:**
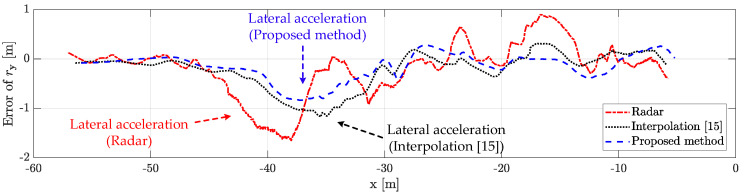
Lateral distance error for scenario-based object estimation: the proposed method outperforms the conventional radar estimation method and the previous interpolation method. When measured with radar, the lateral position error is heavily influenced by the measurement uncertainty. However, there is a lateral position error in all methods due to latency for lateral acceleration via the lane change motion of the object vehicle.

**Figure 13:**
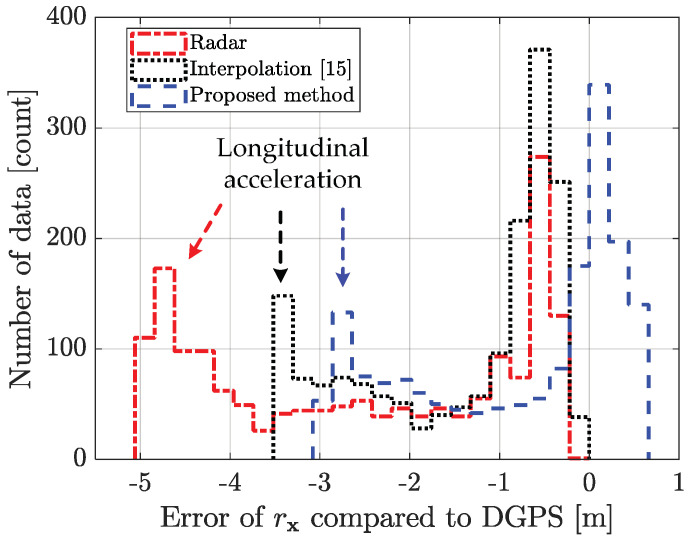
Histogram of the longitudinal distance error for scenario-based object estimation: using the weighted interpolation method improves the estimation performance statistically. However, there is a longitudinal error due to latency for longitudinal acceleration in the acceleration area.

**Figure 14:**
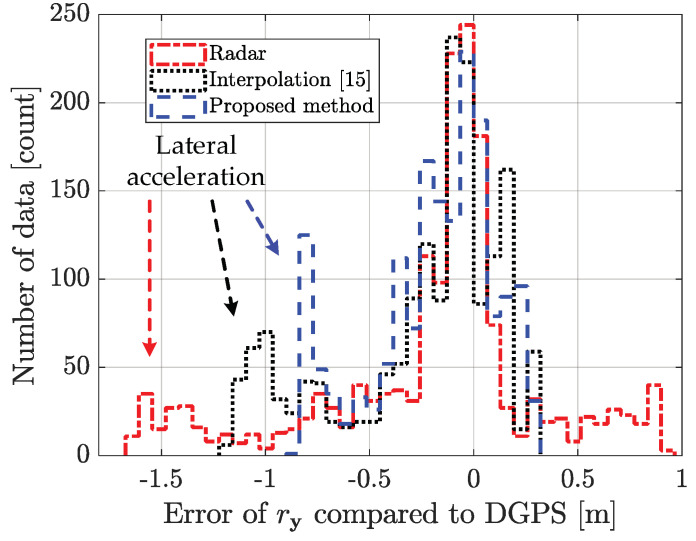
Histogram of the lateral distance error for scenario-based object estimation: using the weighted interpolation method improves the estimation performance statistically. However, there is a lateral position error due to latency for lateral acceleration via the lane change motion of the object vehicle.

The comparative results of the tracking performance are shown in Figure 8. The proposed weighted interpolation scheme improved the object vehicle tracking performance by reducing the estimation error. The scenario-based relative movement of object tracking was plotted in the top view. The proposed weighted interpolation scheme’s performance was similar to that of DGPS. On the other hand, object tracking using a commercial radar had a larger tracking error than the proposed method due to measurement uncertainty and radar latency. Compared to DGPS, the root mean square error (RMSE) of the commercial radar and the proposed method are 3.04 m and 1.29 m in the longitudinal position and 0.57 m and 0.32 m in the lateral position, respectively. When estimated with a commercial radar, there is a longitudinal position error of about −5 m and a lateral position error of about −1.5 m between −43 m and −38 m. This is because the latency significantly affects the longitudinal position error. This error is also affected by the measurement uncertainty and relative acceleration. The influence of relative acceleration will be described in detail in the next paragraph. In addition, there is a lateral position error of about 1 m between −17 m and −15 m. This error is due to the influence of measurement uncertainty. In this regard, Figure 9 shows object tracking in the 3D view, including the relative velocity. The object vehicle changed lanes while increasing speed to overtake the ego vehicle. The proposed method outperforms the conventional radar estimation method and the previous interpolation method [15]. This is because the relative speed was not considered in the previous interpolation method. In this regard, the position error is covered in more detail in the next subsection, with Figure 10, Figure 11, Figure 12, Figure 13 and Figure 14. The proposed weighted interpolation scheme reflects the average position error and covariance for the relative speed, even when there is speed variation. We observed that the proposed weighted interpolation scheme is robust against speed variation and that it outperforms the tracking performance of the commercial radar.

### 5.4. Performance Analysis with Limitation

The proposed method outperforms the conventional radar estimation method and the previously researched interpolation method [15]. The performance for the scenario-based experimental result is shown in Figure 10, Figure 11, Figure 12, Figure 13 and Figure 14. Figure 10 represents the relative longitudinal distance (x-axis) and relative velocity (y-axis) from Figure 9. There are acceleration areas (relative speed increase area) for radar, the previously researched interpolation method, and the proposed method. Figure 11 and Figure 12 show the longitudinal and lateral position errors in terms of the x-axis position. The proposed method has a smaller position error than the conventional radar estimation method and the previous interpolation method compared to DGPS. Previously researched interpolation methods introduce measurement uncertainty and latency errors for speed. This is because speed is not considered. When measured with radar, the lateral position error is heavily influenced by the measurement uncertainty. Figure 13 and Figure 14 show histograms of the relative position error for longitudinal and lateral, respectively. By using the proposed method, longitudinal errors due to latency and lateral errors due to measurement uncertainty are reduced.

However, there is a lateral position error in all methods due to latency for lateral acceleration via the lane change motion of the object vehicle. The estimation performance is improved by using the weighted interpolation method, but there is a limitation to the proposed method. The limitation arises because radar accuracy modeling is used only as the constant relative velocity model (2) and because relative acceleration is not considered. The longitudinal position error increases in the acceleration area, as shown in Figure 11 and Figure 13. This is confirmed to be the effect of latency on relative acceleration. The position error was reduced outside of the acceleration area due to the proposed method. Since the object vehicle’s lane change in the acceleration area is also performed, the lateral position error increases as the relative lateral acceleration increases, as shown in Figure 12 and Figure 14. We have confirmed that the position error occurs in radar, the previously researched interpolation method, and the proposed method due to the influence of relative acceleration.

As future work, research should be conducted to reduce the effects of relative acceleration. The effect of relative acceleration can be reduced by using the relative acceleration model. In this regard, we will further consider the acceleration model using multiple models and expect to improve the collision risk performance using accurate radar estimation.

## 6. Conclusions

This paper proposed a data-driven object vehicle estimation scheme to solve the radar system accuracy problem. For object estimation considering the radar accuracy, we first developed an accuracy model that considers the different error characteristics depending on the zone. The accuracy model was used to solve the measurement uncertainty of radar. We also developed latency coordination for the radar system by analyzing the position error depending on the relative velocity. The developed accuracy modeling and latency coordination methods were applied to object vehicle estimation using weighted interpolation. The utility of the proposed method was validated through a scenario-based estimation experiment. The proposed data-driven object vehicle estimation outperformed the commercial radar algorithm and the previously researched interpolation method. The proposed method is expected to improve object vehicle estimation accuracy. Future work is expected to use an additional acceleration model as multiple models to reduce the effect of relative acceleration. This achievement is critical for autonomous driving technology for developing a high-level controller for functions such as collision risk decision, path planning with collision avoidance, and lane change system. 

## Data Availability

Not applicable.
